# Differential expression of circulating long non-coding RNAs in patients with acute myocardial infarction

**DOI:** 10.1097/MD.0000000000013066

**Published:** 2018-12-21

**Authors:** Zhixiong Zhong, Jingyuan Hou, Qifeng Zhang, Bin Li, Cunren Li, Zhidong Liu, Min Yang, Wei Zhong, Pingsen Zhao

**Affiliations:** a Center for Cardiovascular Diseases; b Guangdong Provincial Engineering; c Center for Precision Medicine; d Meizhou Municipal Engineering and Technology Research Center for Molecular Diagnostics of Cardiovascular Diseases; e Meizhou Municipal Engineering and Technology Research Center for Molecular Diagnostics of Major Genetic Disorders; f Clinical Core Laboratory, Meizhou People's Hospital (Huangtang Hospital), Meizhou Academy of Medical Sciences, Meizhou Hospital Affiliated to Sun Yat-sen University, Meizhou, P.R. China.

**Keywords:** biomarkers, long noncoding RNAs, non-ST-elevation myocardial infarction, peripheral blood mononuclear cells, ST-elevation myocardial infarction

## Abstract

Long noncoding RNAs (lncRNAs) are non-protein coding transcripts regulating various critical physiological and pathological processes, yet limited information is available about lncRNAs expression in acute myocardial infarction (AMI). We aimed to identified differentially expressed lncRNAs in blood samples of patients with AMI to assess their diagnostic value. Differential expression of lncRNAs in peripheral blood mononuclear cells (PBMC) of patients with non-ST-elevation myocardial infarction (NSTEMI) and ST-elevation myocardial infarction (STEMI) was compared by RNA sequencing method and validated by real-time polymerase chain reaction (PCR). The area under the receiver operating characteristic curve (ROC) was used to evaluate diagnostic accuracy. Gene ontology (GO) enrichment and Kyoto Encyclopedia of Genes and Genomes (KEGG) analyses of lncRNA-coexpressed mRNAs were conducted to determine the related biological modules and pathological pathways. RNA sequencing data showed that 58 lncRNAs were differentially expressed between NSTEMI patients and STEMI patients, including 42 upregulated lncRNAs and 16 down-regulated lncRNAs. The ROC curves showed that ENST00000508020.2, LNC_001265, LNC_001526, and LNC_002674 could distinguish AMI patients with preferable sensitivity and specificity. GO enrichment analysis of lncRNA-coexpressed mRNAs indicated that the biological modules were correlated with cell adhesion, calcium ion homeostasis, complement receptor mediated signaling pathway, and immune system process. KEGG pathway analysis indicated that the lncRNAs-co-expressed mRNAs were involved in the regulation of peroxisome proliferators-activated receptors (PPAR) signaling pathway, mTOR signaling pathway, Insulin signaling pathway, HIF-1 signaling, and chemokin signaling pathway. Our results are in line with the previous findings, suggesting that differential expression of lncRNAs would be helpful to understand the molecular mechanism of AMI and might be useful biomarkers for noninvasive diagnostic application. Further studies are still needed to verify our findings and hypothesis.

## Abstract 2

Supplemental Digital Content is available in the text

## 1 Introduction

Acute myocardial infarction (AMI) is a widely common cardiovascular disease and is a predominant cause of fatality globally.^[1,2]^ With the changes in economy situation and life styles, the prevalence of AMI mounting up dramatically year by year in China, which causing a serious public health problem and heavy socioeconomic loss.^[3]^ AMI is typically caused by activation of platelet aggregation at the site of a ruptured or eroded atherosclerotic plaque.^[4,5]^ It is well recognized that age, sex, dyslipidemia, atherosclerosis, hypertension, smoking and diabetes are significantly associated with the risk of AMI.^[6–8]^ Although the relationship between risk factors and AMI has been extensively researched, however, the relation between these 2 remains controversial due to somewhat divergent of the results depending on different clinical subgroups of AMI. Such differences in results are have been speculated that a compound character is derived from a complex interaction of environmental and genetic factors.^[9–12]^

Long noncoding RNAs (lncRNAs) are generally classified as transcripts longer than 200 nucleotides in length and with poor protein coding potential but regulate the expression of coding genes.^[13,14]^ A growing body of evidence suggests that functions of lncRNAs mainly involve in a series of critical physiological and pathological processes through transcriptional or posttranscriptional regulatory mechanisms.^[15–17]^ Some researchers have reported that aberrant expression of lncRNAs in tissues or cells could promote various diseases, such as cancer, autoimmune diseases and cardiovascular diseases.^[18–22]^ More importantly, some studies have indicated that circulating lncRNAs usually present as a secondary structures and are relatively more stable, which facilitate their detection acting as biomarkers in body fluids such as plasma, serum, and urine.^[23,24]^

Many studies have recently been published focusing on the association between adverse cardiovascular and lncRNAs, yet limited information is available about lncRNAs expression in AMI. In the present study, we used RNA-Sequencing method to compare the lncRNAs expression profile difference between non-ST-elevation myocardial infarction (NSTEMI) patients and ST-elevation myocardial infarction (STEMI) patients. The aim of the study was to examine the potential of lncRNAs can serve as novel noninvasive diagnostic biomarkers for differentiating between STEMI and NSTEMI by comparing aberrantly expressed lncRNAs in PBMCs of patients.

## 2 Materials and methods

### 2.1 Study design

Between September 1, 2015, and December 1, 2017, we prospectively enrolled AMI patients from Center for Cardiovascular Diseases at Meizhou People's Hospital (Huangtang Hospital), Meizhou Academy of Medical Sciences, Meizhou Hospital Affiliated to Sun Yat-sen University, China. Based on clinical symptoms, all of the AMI patients were newly diagnosed. The study was performed in accordance with the ethical standards specified by the Declaration of Helsinki and its amendments and approved by the Ethics Committee of Meizhou People's Hospital (Huangtang Hospital), Meizhou Academy of Medical Sciences, Meizhou Hospital Affiliated to Sun Yat-sen University. All participants provided written informed consent before enrollment in the study.

To be eligible, patients needed to have an established coronary disease defined included both NSTEMI and STEMI. The inclusion criteria were as follows: a history of chest pain of at least 15 minutes duration. An increase in activity of the serum cardiac markers, such as creatinine kinase, myohemoglobin, and troponin T. Typical electrocardiographic changes. Exclusion criteria included the following: major surgery or trauma within the previous 3 weeks. Heart failure and severe valvular heart disease. Severe systemic diseases, chronic kidney, hepatic disease, malignant tumor, and inflammatory diseases. Age younger than 18 years. The classifying diagnosis was set at discharge by 1 of 2 experienced cardiologists.

### 2.2 Sample preparation and total RNA extraction

Peripheral blood anticoagulated by ethylene diamine tetraacetic acid (EDTA) were collected shortly after the patients were admitted to hospital within 2 hours after onset of chest pain. Human peripheral blood mononuclear cells (PBMC) was isolated using Lymphoprep (Axis-Shield, Norway) and then carefully transferred into an RNase-free tube for extraction of RNA. Total RNA was processed through TRIzol reagent in accordance with the manufacturer's instructions (Invitrogen, CA). The concentration of total RNA was assessed by Nanodrop 2000 (Thermo Scientific, Waltham, MA) and the integrity of the RNA of each sample was analyzed using standard denaturing agarose gel electrophoresis. The individual RNA samples were stored at –80 °C until further use.

### 2.3 RNA sequence and data processing

LncRNA sequence profiling was performed using Illumina Hiseq 4000 platform according to the protocols in ShenZhen Realomics Inc. In brief, 3 μg of total RNAs were depleted of ribosomal RNA using the Epicentre Ribo-zeroTM rRNA Removal Kit (Epicentre, Madison, WI). Eluted RNA was prepared for sequencing using Illumina protocols, and then sequenced on the Hiseq 4000 platform (Illumina) to generate 150 bp paired-end reads. The raw RNA-seq reads were aligned and mapped using TopHat v2.0.9, and transcriptome assemblies were generated using Cufflinks v2.1.1 with the default parameters. Normalized expression data were subsequently analyzed for differently expressed lncRNAs and protein-coding genes using the Cuffdiff v2.1.1. Differentially expressed lncRNAs and mRNAs were identified through fold change filtering at a threshold values of >2 and <–2 fold change under false discovery rate (FDR) protection (*P* < .05).

### 2.4 Construction of the lncRNA–mRNA coexpression network

To identify the relationship between lncRNAs and mRNAs, the lncRNA–mRNA coexpression network was constructed based on correlation analysis between the differential expressed lncRNAs and mRNAs. We selected the part that Pearson correlation coefficients, ≥0.95, to generate the coexpression network using the open source bioinformatics software Cytoscape (v3.4, the Cytoscape Consortium, San Diego, CA). Associations between lncRNAs and mRNAs were connected by solid lines to build the lncRNA–mRNA coexpression network, diamond represents as lncRNAs and circle represents as mRNAs.

### 2.5 Gene ontology and pathway analysis

Gene ontology (GO) analysis and signaling pathway analysis was applied to determine the potential functions roles of differentially expressed mRNAs base on the Database for Annotation, Visualization and Integrated Discovery (DAVID; http://david.abcc.ncifcrf.gov) and Kyoto Encyclopedia of Genes and Genomes (http://www.genome. jp/kegg/) database. Significance is judged when *P* value is <.05.

### 2.6 Validation by real-time quantitative PCR

cDNA was synthesized and Real-Time Quantitative PCR (RT-qPCR) of lncRNAs expression level was performed using Luna Universal One-Step RT-qPCR kits (New England Biolabs, MA) according to the manufacturer's protocol. The PCR were performed on the LightCycler 480/II machine (Roche, Mannheim, Germany) and the PCR conditions were set as follow: 1 cycle of 10 minutes at 55 °C, 1 cycle of 1 minute at 95 °C, 45 cycles of 10 second at 95 °C and 30 seconds at 60 °C, and 1 cycle of 1 minute at 60 °C finally. Gene expression levels were quantified were calculated using the method of 2^−ΔΔct^ and normalized to the internal control of the expression of glyceraldehyde-3-phosphate dehydrogenase (GAPDH).^[25]^ The level of each lncRNAs in STEMI patients was expressed as the fold changes against the averaged level of the same lncRNA in NSTEMI patients.

### 2.7 Statistical analysis

Data were analyzed using SPSS software version 19.0 (SPSS Inc, Chicago, IL). Differential expression levels of lncRNAs were compared via independent-sample *t* tests between 2 groups. Receiver operating characteristic (ROC) curve was constructed to evaluate the predictive power of circulating lncRNAs between the STEMI and NSTEMI patients, and area under the curve (AUC) was used to assess the diagnostic values of lncRNAs. GO and pathway analyses were evaluated using Fisher exact test. Statistical significance was defined as *P* <.05.

## 3 Results

### 3.1 The patients’ clinical characteristics

A total of 7 NSTEMI patients and 7 STEMI patients were recruited for the lncRNA sequence analysis in this study. The clinical characteristics of the enrolled patients are summarized in Table 1. There was no significant difference in the distribution of sex, smoking, drinking, hypertension, diabetes mellitus, and dyslipidemia between NSTEMI group and STEMI group (*P* > .05). Meanwhile, there were no significant differences was found in age, systolic blood pressure (BP), diastolic BP, total cholesterol (TC), triglycerides (TG), high density lipoprotein cholesterol (HDL-C), low density lipoprotein cholesterol (LDL-C), cardiac troponin I (cTnI), brain natriuretic peptide (BNP) between the 2 groups (*P* > .05). Another a total of 20 NSTEMI patients and 20 STEMI patients were also enrolled for the quantitative real time polymerase chain reaction (qRT-PCR) validation and ROC curve analysis. The baseline clinical characteristics of the study subjects are shown in Table 1. There no statistical differences were observed excepted systolic BP (*P* = .010) and cTnI (*P* = .022) between NSTEMI group and STEMI group.

**Table 1:**
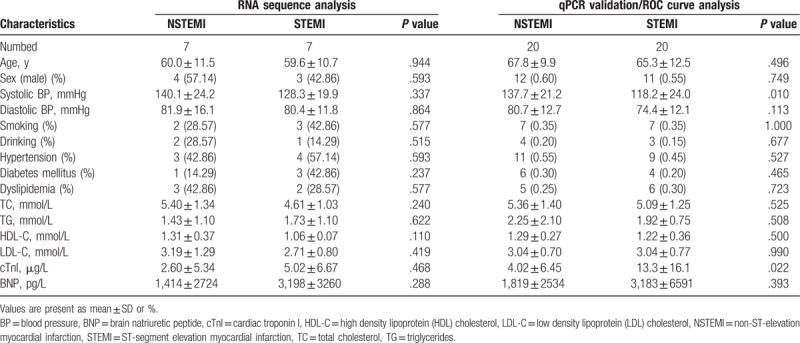
Clinical characteristics of patients enrolled in our study.

### 3.2 Differentially expressed lncRNAs and mRNAs in PBMCs

**Figure 1:**
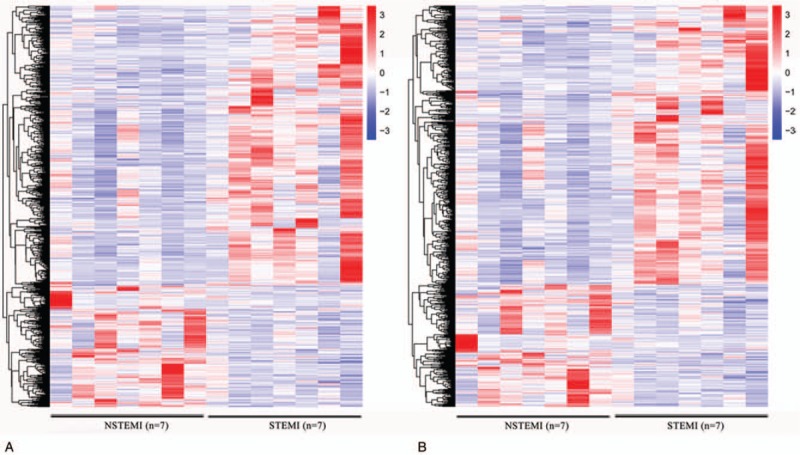
Expression profiles of differentially expressed lncRNAs and mRNAs in NSTEMI and STEMI patients. Differentially expressed lncRNAs and mRNAs between NSTEMI and STEMI patients were subjected to hierarchical clustering. Red color indicates high relative expression and blue color indicates low relative expression. (A) lncRNA; (B) mRNA. lncRNAs = long noncoding RNAs, NSTEMI = non-ST-elevation myocardial infarction, STEMI = ST-elevation myocardial infarction.

To systematically investigate the expression levels of lncRNAs and mRNAs associated with AMI, RNA sequencing method was used to characteristic the profiles of PBMC of 7 NSTEMI patients and 7 STEMI patients. Red color indicates high relative expression and blue color indicates low relative expression, respectively. The results of hierarchical clustering showed systematic variations in the expression of lncRNAs (Fig. 1A) and mRNAs (Fig. 1B) between the 2 groups. These observations suggest that a potential differentiate STEMI from NSTEMI by a difference in the expression profile of either lncRNAs or mRNAs.

**Figure 2:**
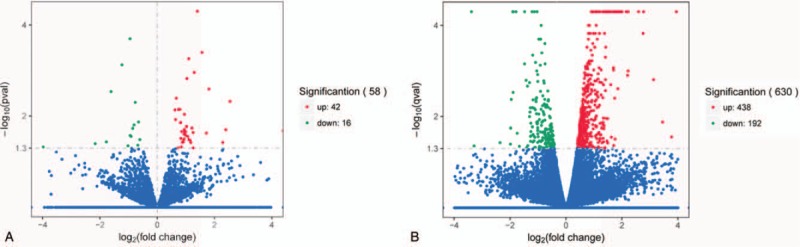
Volcano plot showing differentially expressed genes lncRNAs and mRNAs (STEMI patients vs NSTEMI patients). (A) Differentially expressed lncRNAs, and (B) mRNAs. The left vertical line stands for the log_10_*P*-value and the horizontal line corresponds to the log_2_ fold change value. The red dots and green dots represent the significantly differentially expressed genes (*P* ≤ .05), while the blue dots are not statistically significant (*P* > .05). lncRNAs = long noncoding RNAs, NSTEMI = non-ST-elevation myocardial infarction, STEMI = ST-elevation myocardial infarction.

A scatterplot was a visualization used to assess lncRNAs and mRNA expression variation between patients with NSTEMI and STEMI control, respectively (Fig. 2). The red dot indicates the genes that are upregulated and green color indicates the genes that are down-regulated, respectively. A total of 58 lncRNAs were found to be significantly differentially expressed with FC ≥1 (*P* < .05), among which 42 lncRNAs were upregulated and 16 lncRNAs were down-regulated in the STEMI patients, compared with the NSTEMI patients (Fig. 2A). Additionally, 438 mRNAs were upregulated, and 192 were down-regulated in the STEMI patients compared with the NSTEMI patients (Fig. 2B). The top 10 upregulated and 10 down-regulated lncRNAs and mRNAs between 2 groups were listed in Tables 2 and 3, respectively.

**Table 2:**
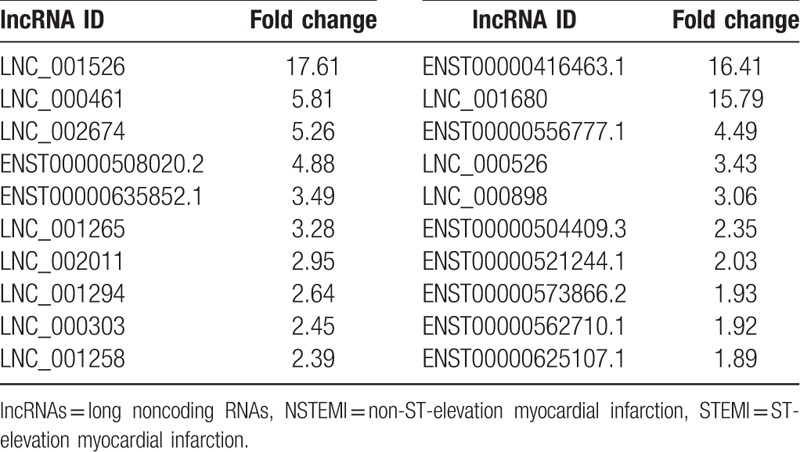
The top 10 of differentially expressed lncRNAs according to the fold change (FC) values in STEMI patients compared with NSTEMI patients.

**Table 3:**
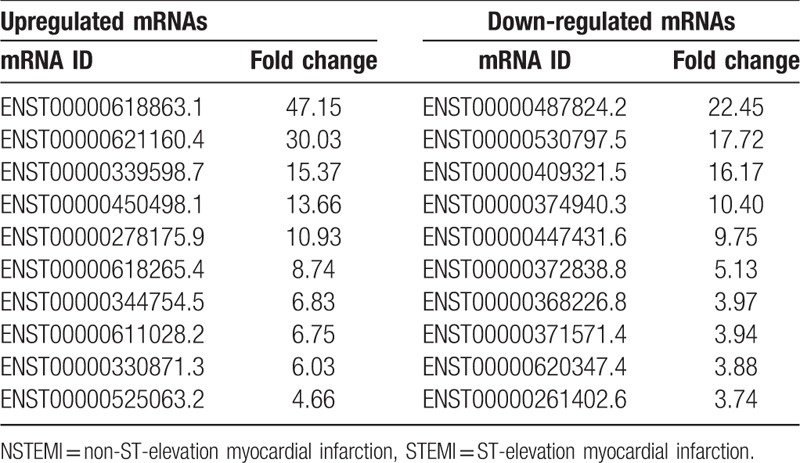
The top 10 of differentially expressed mRNAs according to the fold change (FC) values in STEMI patients compared with NSTEMI patients.

### 3.3 lncRNA–mRNA coexpression network

**Figure 3:**
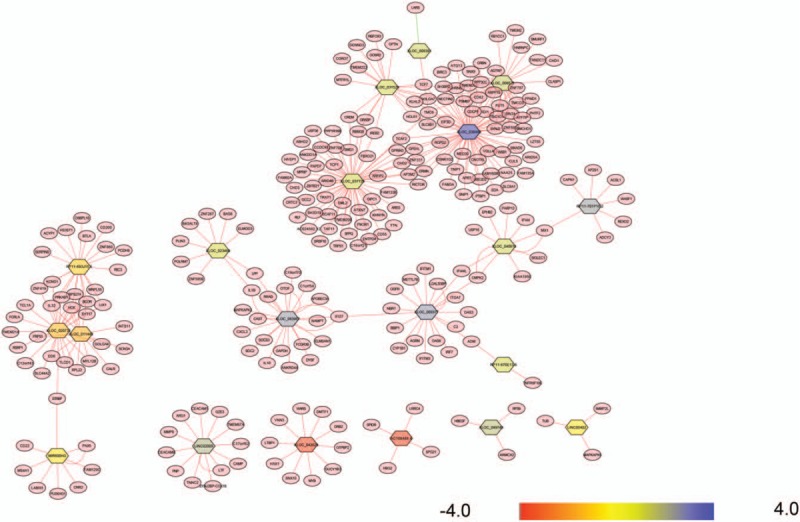
LncRNA–mRNA-network was constructed based on the correlation analysis between the differentially expressed lncRNAs and mRNAs. Associations between lncRNAs and mRNAs were connected by solid lines to build the lncRNA–mRNA coexpression network, diamond represents as lncRNAs and circle represents as mRNAs. lncRNAs = long noncoding RNAs.

Coexpression network analysis was constructed between the dysregulated expressed lncRNAs and mRNAs. In total, 32 lncRNAs and 268 mRNAs were included in the coexpression network. Our data showed that the coexpression network was composed of 273 network nodes and 336 connections, as shown in Fig. 3. The coexpression network indicated that 1 mRNA may correlate with 1 to 4 lncRNAs, and 1 lncRNA may correlate with 1 to 64 mRNAs. The details of the source lncRNA and their corresponding genes are presented in File S1.

### 3.4 Gene enrichment and pathway analysis

**Figure 4:**
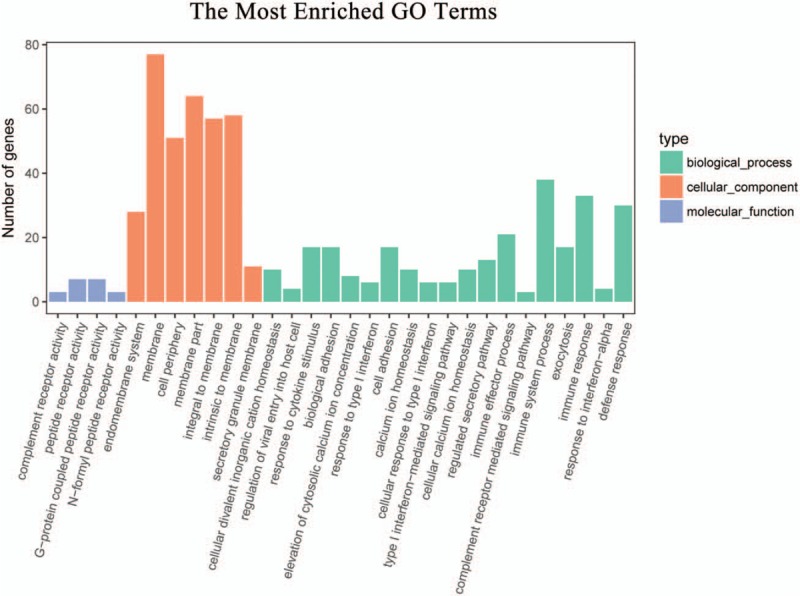
Gene enrichment analysis of significantly correlated mRNAs targets of lncRNAs. The ontology covered 3 domains: biological process, cellular component, and molecular function. lncRNAs = long noncoding RNAs.

**Figure 5:**
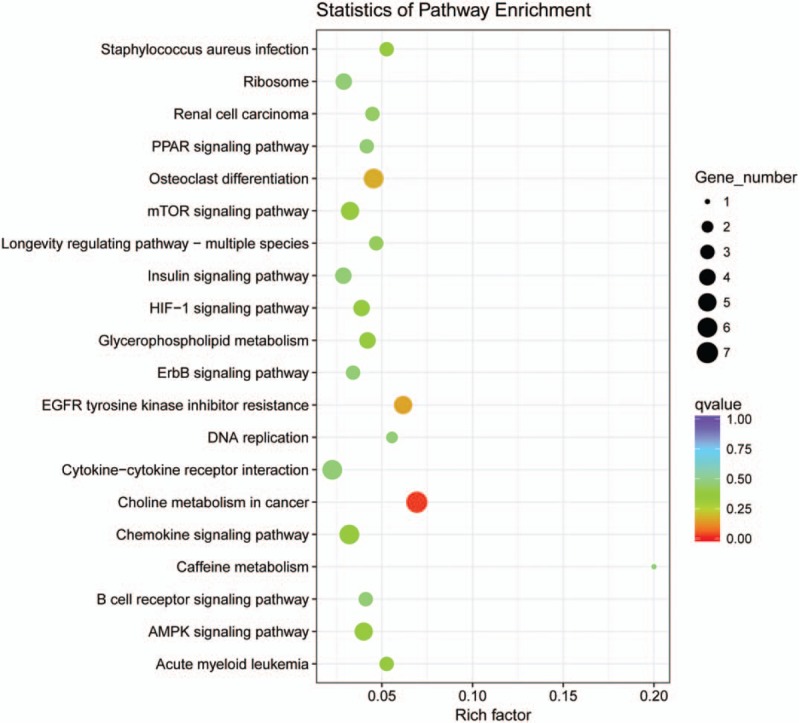
Scatter plot of KEGG pathway enrichment statistics. An *q* values <0.05 and were considered as significantly enriched pathways.

A GO enrichment analysis was performed to determine the function of the coexpressed mRNAs identified in this study. Genes were organized into hierarchical categories to uncover gene regulatory networks enrichment in biological processes, cellular components, and molecular functions (Fig. 4). Among the genes corresponding to the identified mRNAs, 2249 are involved in biological processes, 292 in cellular components, and 352 in molecular functions. We found that these lncRNAs-coexpressed mRNAs were associated with cell adhesion, calcium ion homeostasis, complement receptor mediated signaling pathway and immune system process (ontology: biological process), peptide receptor activity (ontology: molecular function), and cell periphery (ontology: cellular component). KEGG pathway analysis indicated that the lncRNAs-coexpressed mRNAs were involved in the regulation of PPAR signaling pathway, mTOR signaling pathway, insulin signaling pathway, HIF-1 signaling, and chemokine signaling pathway (Fig. 5).

### 3.5 qRT-PCR validation of lncRNAs expression

**Figure 6:**
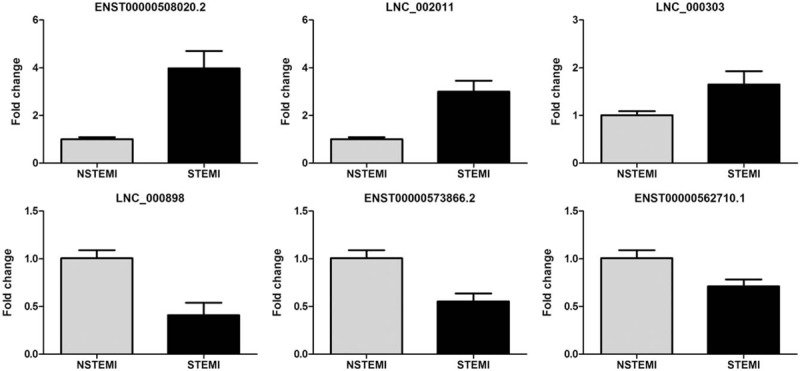
Expression level of differentially expressed lncRNAs in NSTEMI and STEMI patients. ENST00000508020.2, LNC_002011, LNC_000303 LNC_000898, ENST00000573866.2, and ENST00000562710.1 was detected by qPCR and normalized by GAPHD expression in STEMI patients and NSTEMI patients (All *P* values were less than .05). lncRNAs = long noncoding RNAs, NSTEMI = non-ST-elevation myocardial infarction, STEMI = ST-elevation myocardial infarction.

Based on the fold change, significance, and number of transcripts, 3 upregulated lncRNAs (ENST00000508020.2, LNC_002011, and LNC_000303) and 3 down-regulated lncRNAs (LNC_000898, ENST00000573866.2, and ENST00000562710.1) were randomly selected as candidates for further validation in an additional AMI samples. We verified the expression of these lncRNAs by qRT-PCR using GAPDH as the reference gene with the 2^-ΔΔCT^ method. The primer sequences are presented in Table 4. Log2-transformed fold changes and dot plots of expression in 20 STEMI patients versus 20 NSTEMI patients are shown in Fig. 6. A general consistency between the real-time PCR and with the RNA sequencing data was confirmed in all selected lncRNAs in terms of regulation direction.

**Table 4:**
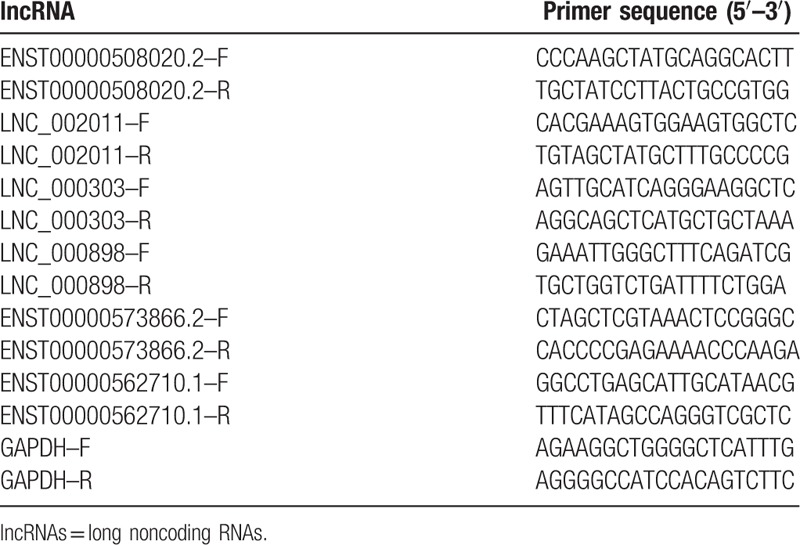
Primers used in qRT-PCR.

### 3.6 ROC curve analysis

**Figure 7:**
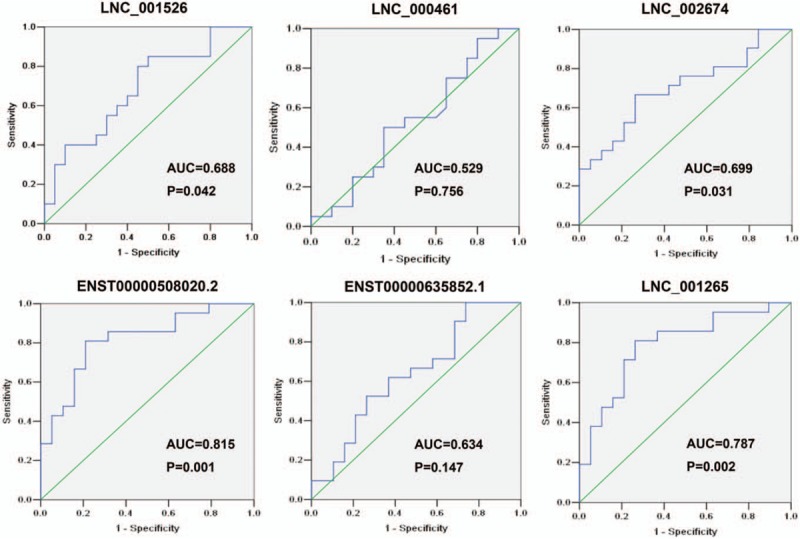
The receiver operating characteristic (ROC) curve analysis for discriminative ability between STEMI patients and NSTEMI patients by the 6 lncRNAs (LNC_001526, LNC_000461, LNC_002674, ENST00000508020.2, ENST00000635852.1 and LNC_001265). lncRNAs = long noncoding RNAs, NSTEMI = non-ST-elevation myocardial infarction, STEMI = ST-elevation myocardial infarction.

Based on the above findings, more attention was paid if these lncRNAs could work on distinguishing between STEMI and NSTEMI patients. Among the upregulated lncRNAs, ROC analysis was created to confirm the diagnostic value of lncRNAs in 20 NSTEMI patients and 20 STEMI patients and AUC was generated to evaluate the diagnostic values of the 6 selected lncRNAs (FC > 3). The AUC and *P* values are summarized in Fig. 7. The AUC values of these lncRNAs for differentiating between patients with 20 NSTEMI patients and 20 STEMI patients were range from 0.529 to 0.815.

## 4 Discussion

With the developing of social economy and the changing of life styles, AMI has been a severe cardiovascular disease worldwide and exerts huge burdens in China.^[1,3]^ It is clear that AMI is a complex and multifactorial disorder that results from the complex interaction between inherited genetic and environmental factors.^[9,10]^ However, the detailed molecular mechanisms of genetic of AMI are still largely unknown. LncRNAs had long been considered serve as valuable biomarkers in a number of human diseases, which may be used for clinical application. There is limited knowledge of lncRNAs regulation in human peripheral blood or their role in cardiovascular diseases. In addition, circulating RNAs in serum, plasma, or other body liquid have emerged as useful and highly promising biomarkers for noninvasive diagnostic application.^[23,24]^ An early and timely diagnosis engages for an immediate initiation of reperfusion therapy to reduce the AMI mortality rate largely. In this study, we identified 58 lncRNAs and 630 mRNAs abnormally expressed in PBMC of STEMI patients compared with NSTEMI participants. Our qRT-PCR results showed that the levels of ENST00000508020.2, LNC_002011, LNC_000303, LNC_000898, ENST00000573866.2, and ENST00000562710.1 expression were highly consistent with the RNA sequencing data. Further ROC curve analysis indicated that the AUC as well as the sensitivity and specificity of the selected may serve as circulating biomarkers for the diagnosis of AMI. Overall, our results demonstrate that lncRNAs have a probable role in AMI development and progression and may serve as a biomarker for the earlier diagnosis of AMI.

In general, many cardiovascular and cerebrovascular diseases can be attributed to a growing number of identified genetic alterations, that contribute to the pathogenesis and progression of atherosclerosis.^[8,9]^ Over the past few years, studies on lncRNAs have become common in cardiovascular biology research and altered lncRNAs profiles have been identified in cardiac development, atherosclerosis, myocardial infarction, heart failure, aneurysms, and hypertension, indicating that aberrant expression of certain lncRNAs contributes to cardiovascular diseases.^[13–15,26,27]^ Despite the considerable progress in understanding lncRNAs that has accompanied over a decade of research, studies of lncRNAs in the cardiovascular system are still in their infancy, particularly with regard to AMI.

Following the exploration in expression pattern of lncRNAs in some types of cardiovascular disease including AMI in other populations, we performed a comprehensive analysis profile of lncRNAs in Hakka ethnicity in China. Using RNA sequencing analysis, expression profiles of lncRNAs and mRNAs in 7 NSTEMI patients and 7 STEMI patients were studied. A total 42 up- and 16 down-regulated lncRNAs were identified to be significantly and differentially expressed in STEMI patients and accordingly in mRNA levels, 438 mRNAs were upregulated and 192 mRNAs were down-regulated when compared with that in NSTEMI patients. Dysregulated expression or function of lncRNAs has been recognized to contribute to heart development and complex cardiovascular diseases, as well as AMI.^[10,11]^ The lncRNA *UCA1* (urothelial carcinoma-associated 1), proposed as a biomarker as well, presented altered expression in MI patients.^[28]^ A recent study provided evidence that variants in *ANRIL* gene correlated with its expression contribute to myocardial infarction risk.^[29]^ In a mouse model of MI induced by coronary ligation, several lncRNAs were dysregulated in the heart, among which the 2 most strongly upregulated were named *Mirt1* and *Mirt2* (myocardial infarction-associated transcript 1 and 2).^[30]^

LncRNAs were shown to modulate gene expression at the epigenetic, transcriptional, and posttranscriptional levels. They are involved in many diverse biological and pathological processes such as genomic imprinting, cell differentiation and proliferation, chromatin modification, cell fate determination, and thus facilitate or suppress the development of human diseases.^[31,32]^ In our study, GO analysis of the lncRNAs-coexpressed mRNAs showed the significantly changed GO terms were mainly involved in cell adhesion, calcium ion homeostasis, complement receptor mediated signaling pathway, and immune system process, which might be important for the pathological process of myocardial infarction.^[33,34]^ Pathway analysis identifying the enriched pathways corresponding to the differentially expressed lncRNAs showed that some of them were involved in the regulation of PPAR signaling pathway, mTOR signaling pathway, insulin signaling pathway, HIF-1 signaling, and chemokin signaling pathway.^[10,35,36]^ These findings provide useful bioinformation with functional links, which may have a potential role in AMI occurrence and development. Previous study have reported that inflammation and myocardial apoptosis are key pathological processes involved in myocardial ischemia and reperfusion injury via prompting a release of cytokines, oxygen free radicals, and other pro-inflammatory.^[30,37–40]^ Several other observations also provide evidences that coincided with the previous studies and reinforced the veracity of our results.^[41–44]^ However, more work needs to be done to further characterize the lncRNAs function during the process of AMI.

Circulating LncRNAs have emerged as non-invasive diagnostic biomarkers. In this study, we also investigated the potential utility of circulating LncRNAs as diagnostic biomarkers of AMI (Fig. 7). Among which, it is noteworthy that the AUC values of ENST00000508020.2 and LNC_001265 showed high accuracy in discriminating STEMI patients and NSTEMI patients, with an AUC of 8.815 (*P* = .001) and 0.787 (*P* = .002), respectively. Moreover, LNC_001526 (AUC = 0.688, *P* = .042) and LNC_002674 (AUC = 0.699, *P* = .031) had the modest efficiency in distinguishing STEMI patients and NSTEMI patients. The results suggested that these LncRNAs might serve as promising biomarkers for AMI patients. However, greater sample sizes are needed to further confirm the potential applications of these LncRNAs as diagnostic tool for diagnosis of AMI.

Our study still has limitations. As the sample size for RNA sequencing analysis and verification were small and only tested in 1 ethnic group, therefore, its validity should be tested further in a large population. Thus, the present study should be considered as hypothesis generating, further genetic and experimental investigations involving target genes is planned.

In summary, the present study using RNA sequencing approach to examine the expression profile of lncRNA in PBMC from STEMI patients in comparison with NSTEMI patients. Results provide previously unreported bioinformation on lncRNAs expression and corresponding mRNAs expression in AMI patients. This finding would be helpful to understand the molecular mechanism of AMI and future analysis will focus on whether lncRNAs may serve as a potential noninvasive diagnostic for AMI.

## Acknowledgments

The authors would like to thank other colleagues whom were not listed in the authorship of Clinical Core Laboratory and Center for Precision Medicine, Meizhou People's Hospital (Huangtang Hospital), Meizhou Academy of Medical Sciences, Meizhou Hospital Affiliated to Sun Yat-sen University for their helpful comments on the manuscript.

## Author contributions

Pingsen Zhao conceived and designed the experiments; Zhixiong Zhong, Jingyuan Hou, Qifeng Zhang, Bin Li, Cunren Li, Zhidong Liu, Min Yang, and Wei Zhong recruited subjects, collected clinical data and conducted the laboratory testing. Pingsen Zhao and Jingyuna Hou prepare the manuscript. Pingsen Zhao reviewed the manuscript.

**Conceptualization:** Pingsen Zhao.

**Data curation:** Cunren Li, Wei Zhong, Pingsen Zhao.

**Formal analysis:** Jingyuan Hou, Zhidong Liu, Min Yang, Wei Zhong, Pingsen Zhao.

**Funding acquisition:** Jingyuan Hou, Pingsen Zhao.

**Investigation:** Zhixiong Zhong, Jingyuan Hou, Bin Li, Cunren Li, Zhidong Liu, Pingsen Zhao.

**Methodology:** Zhixiong Zhong, Jingyuan Hou, Qifeng Zhang, Bin Li, Cunren Li, Zhidong Liu, Min Yang, Pingsen Zhao.

**Project administration:** Pingsen Zhao.

**Resources:** Zhixiong Zhong, Jingyuan Hou, Qifeng Zhang, Bin Li, Cunren Li, Zhidong Liu, Min Yang, Wei Zhong, Pingsen Zhao.

**Software:** Zhixiong Zhong, Jingyuan Hou, Qifeng Zhang, Bin Li, Min Yang, Wei Zhong, Pingsen Zhao.

**Supervision:** Zhixiong Zhong, Pingsen Zhao.

**Validation:** Zhixiong Zhong, Jingyuan Hou, Qifeng Zhang, Pingsen Zhao.

**Visualization:** Pingsen Zhao.

**Writing – original draft:** Zhixiong Zhong, Jingyuan Hou, Pingsen Zhao.

**Writing – review & editing:** Pingsen Zhao.

## Supplementary Material

Supplemental Digital Content
